# Irisin, physical activity and fitness status in healthy humans: No association under resting conditions in a cross-sectional study

**DOI:** 10.1371/journal.pone.0189254

**Published:** 2018-01-30

**Authors:** Nathalie Biniaminov, Susanne Bandt, Alexander Roth, Sascha Haertel, Rainer Neumann, Achim Bub

**Affiliations:** 1 Institute of Sports and Sports Science, Karlsruhe Institute of Technology, Karlsruhe, Germany; 2 Department of Physiology and Biochemistry of Nutrition, Max Rubner-Institut, Federal Research Institute of Nutrition and Food, Karlsruhe, Germany; Medical Clinic, University Hospital Tuebingen, GERMANY

## Abstract

Regular physical activity and physical fitness are closely related to a positive health status in humans. In this context, the muscle becomes more important due to its function as an endocrine organ. Muscle tissue secretes “myokines” in response to physical activity and it is speculated that these myokines are involved in physical activity induced positive health effects. Recently, the newly discovered myokine Irisin thought to be secreted by the muscle in response to physical activity and might be related to the health inducing effect by inducing browning of white adipose tissue. Speculating that myokines at least partly mediate exercise related health effects one would assume that regular physical activity and physical fitness are associated with resting Irisin concentrations in healthy humans. To investigate the association between resting Irisin concentration and either short-term physical activity, habitual physical activity, or physical fitness, data of 300 healthy participants from the cross-sectional KarMeN-study were analyzed. By applying different activity measurements we determined short-term and habitual physical activity, as well as physical fitness. Fasting serum samples were collected to determine resting Irisin concentrations by Enzyme-linked Immunosorbent Assay.

Multivariate linear regression analysis served to investigate associations of the individual physical activity parameters with Irisin concentrations. Therefore, lean body mass and total fat mass (both determined by dual-energy X-ray absorptiometry) as well as age and parameters of glucose metabolism were included as confounders in multivariate linear regression analysis. Results showed that Irisin serum concentrations were not related to measures of physical activity and physical fitness in healthy humans under resting conditions, irrespective of the applied methods. Therefore we assume that if physical activity related effects are partly induced by myokines, permanently increased Irisin serum concentration may not be necessary to induce health-related exercise effects.

## Introduction

Regular physical activity (PA) and physical fitness (PF) are closely related to a positive health status in humans [1, 2]. In contrast, being inactive increases the risk of developing chronical disease [3, 4]. Several mechanisms are involved in the health inducing effect of exercise [5]. In this context, the muscle seems to play an important role as a secretory organ [6]. The substances that are supposed to be secreted by the muscles are named “myokines” [7]. Myokines seem to be secreted by the muscle in response to PA [8] and they are assumed to be involved in PA induced positive health effects [9] by generating their effects in an auto-, para-, or endocrine manner [10].

Within the last few years, rising attention has been given to the myokine Irisin. Its secretion into the circulation occurs after transcription from its fibronectin type III domain containing protein 5 gene and proteolytical cleavage at the extracellular surface of cells [11]. Fibronectin type III domain containing protein 5 gene expression mainly takes place in muscle tissue and to a lesser degree in other tissues, such as adipose tissue [12]. Its secretion thought to be triggered by the peroxisome proliferator-activated receptor (PPAR) gamma coactivator 1 alpha (PGC-1 alpha), whose expression in turn is induced by PA [13–15]. It is speculated that Irisin induces browning of white adipose tissue. Browning describes the development of multilocular, uncoupling protein 1 positive adipocyte inside fat tissue, which is supposed to have a beneficial impact on energy expenditure and whole body homeostasis [11, 16–18]. Due to the postulated browning inducing effect, Irisin might be an important target for reducing the risk of developing overweight associated diseases [19]. It is therefore a major target in current research to identify Irisin as a mediator of the PA inducing health effects.

Results from current studies focusing on the influence of PA on circulating Irisin are inconsistent. While most studies show an impact of acute PA on Irisin blood concentrations [20–29], results on the association between PA status [20, 30], and PF [25, 26, 31, 32] on resting Irisin concentrations are inhomogeneous. Additionally, results of intervention studies investigating a given workload or time of intervention on circulating Irisin are also inconsistent [22, 24, 26, 30, 32–36].

The inhomogeneity of the results of current data might be related to differences in study characteristics, e.g. study designs and number and type of tests of determining PA and Irisin concentration. Speculating that myokines at least partly mediate exercise related health effects one would assume that regular PA and PF are associated with resting Irisin concentrations in healthy humans.

Therefore, the aim of this study was to obtain a comprehensive overview of the PA status by determining different markers of short-term and habitual PA as well as PF in a well described cohort of healthy humans under strictly controlled conditions and thereby investigating the association between resting Irisin concentrations in serum and PA status and PF.

## Material and methods

### Participant characteristics and study design

For the present analysis, data from the cross-sectional Karlsruhe Metabolomics and Nutrition (KarMeN)-study were used [37]. The study was conducted between March 2012 and July 2013 at the Human Studies Division of the Max Rubner-Institut in Karlsruhe, Germany. The aim of KarMeN was to investigate the associations of the metabolome with major lifestyle factors, like nutrition and PA and the health status of healthy man and women of different ages. After the recruitement process a total of 301 healthy male and female participants aged 18–80 years remained. Health status was assessed by collecting medical history data and conducting basic physical examination. Volunteers suffering from acute or chronic therapy requiring diseases were excluded. Details on inclusion and exclusion criteria and detailed information on the study design are published elsewhere [37]. Briefly, all of the participants were required to visit the study center for a total of three times and underwent a series of examinations, e.g. fasting blood sampling, indirect calorimetry, and cardiopulmonary exercise testing. Each examination day followed an identical schedule and the examinations were conducted following standard operating procedures. For our research we had to exclude one subject because of missing myokine data. Therefore data of 300 healthy participants served to investigate the association between fasting serum Irisin concentrations and short-term, habitual PA, and PF.

### Ethics and dissemination

The study has been performed in accordance with the Declaration of Helsinki. It was registered at the German Clinical Trials Register (No: DRKS00004890) and approved by the Ethics Committee of the State Medical Chamber of Baden-Württemberg, Stuttgart, Germany (F- 2011- 051). Approval for dual-energy X-ray absorptiometry (DXA) measurements in healthy participants was obtained from the Federal Office for Radiation Protection (Z5- 22462/2-2011-043). All participants were informed in detail about the examinations, procedures, and measurements and gave their written consent. Confidentiality of personal data was guaranteed by following the regulations of the State Medical Chamber and the Federal Data Protection Act.

### Sample processing, storage, and Irisin analysis

For Irisin and insulin analysis fasting serum samples were collected. After collection, the samples were immediately centrifuged (1850g by 4C°), aliquoted into cryo vials, and stored in the gas phase of liquid nitrogen (below -190°C). To determine the coefficient of variability, an internal quality control sample was prepared from pooled serum samples. The coefficient of variability served to express the repeatability and precision of the test. Blood samplings were placed around the activity measurements, to combine relevant parameters for metabolic biostatistical analysis. See the study design for more details published elsewhere [37]. Irisin and insulin serum concentrations were determined by Enzyme-linked Immunosorbent Assays (ELISA) (Irisin: EK-067-29 from Phoenix Pharmaceuticals, Karlsruhe, Germany; Insulin: ME E-0900 from LDN, Nordhorn,Germany). All analyses were implemented according to the manufacturer’s instructions. For Glucose and HbA1c, blood were analyzed by a certified clinical chemistry laboratory (MZV Labor PD Dr Volkmann, Karlsruhe, Germany).

### Anthropometry

Body height (in cm) and body weight (in kg) was measured in underwear and without shoes using a standardized scale and stadiometer from Seca (Seca 285, Hamburg, Germany). Body mass index (BMI) was calculated by dividing the weight (kg) by square of height in metres (m^2^).

Body composition was measured using the GE Lunar iDXA (GE Healthcare, Munich, Germany). To ensure minimal x-ray absorption by clothing or other objects, participants were asked to lie on the scanner table only wearing underwear. Participants positioned themselves on the table according to the instructions of the study nurse. Lean body mass (LBM) and fat mass (FM) were calculated with the supplementary software Body Composition. FM in percent was described as percentage of the total body weight estimated by DXA.

### Resting energy expenditure

Resting energy expenditure (REE) was analyzed by indirect calorimetry using Vmax™ Encore indirect calorimeter (Vmax-Serie 29n, CareFusion Germany 234 GmbH, Hoechberg, Germany). The measurements were performed according to the manufacturer's instructions. The oxygen uptake and carbon dioxide exhalation were measured with a canopy while the participants were lying in a quiet, thermo-neutral room. All measurements were performed in fasting condition (from 10 p.m. the day before). REE was calculated by the Vmax™ Encore software (version v21-2A) and is indicated in kcal per 24 hours.

### Physical activity and fitness assessment

To cover aspects of PA as well as PF, we applied three different activity assessment tools each for a specific time period and categorized the outcome parameters into three groups: short-term PA, habitual PA and PF (Table 1).

**Table 1:** Physcial activity categories and the corresponding examinations and outcome parameters.

Categories	Examinations	Outcome parameters	Outcome parameters representing PA categories	
Short-term physical activity	Accelerometry	AEE	AEE	TEE
	Indirect calorimetry	REE		
	Thermogenesis ^a^	DIT		
	TEE / REE		PAL	
Habitual physical activity	International Physical Activity Questionnaire		MET	
Physical fitness	Cardiopulmonary exercise test		VO_2 peak_	
	Cardiopulmonary exercise test and lactate test		P_IAT_	

AEE, activity energy expenditure; REE, resting energy expenditure; DIT, diet-induced thermogenesis; TEE, total energy expenditure; PAL, physical activity level; MET, metabolic equivalent of task; VO_2 peak_, peak oxygen consumption; P_IAT_, power at individual anaerobic threshold.

^a^ 10% of TEE for diet-induced thermogenesis

### Short-term physcial activity

The level of short-term PA was analyzed over a period of seven consecutive days using Actiheart^®^ (CamNtech, Cambridge, UK), a device that combines heart rate monitoring and an uniaxial accelerometer to assess movement. Actiheart^®^ is waterproof and weighs 10 g. Participants were instructed to wear Actiheart^®^ during all activities day and night (with the exception of visiting the sauna or solarium and while diving). The device was attached with electrodes (Red Dot™ EKG Elektrode 2271, 3M™, Neuss, Germany) on the left lower chest. In this study Actiheart^®^ was set up to record heart rate and movement counts with a time interval of 15 s. Data from Actiheart^®^ devices were downloaded after measurement and analyzed using the supplied software (version 4.0.103, CamNtech, Cambridge, UK). This provided estimates of the activity energy expenditure (AEE) in kcal per day. The mean total energy expenditure (TEE) in kcal per day was calculated with AEE measured by Actiheart^®^, REE measured by indirect calorimetry, and diet-induced thermogenesis (assuming 10% of TEE). Physical activity level (PAL) was calculated by dividing calculated TEE by measured REE.

### Habitual physical activity

To determine the habitual PA of the last three months, the participants were asked to fill out the international physical activity questionnaire (IPAQ). We applied the long-form of the IPAQ that covers frequency and duration of each activity, such as walking, moderate and vigorous activities in each of the following domains: work, active transportation, leisure-time, domestic, and garden. Only sustained activities of more than 10 minutes should be noted. In accordance with the guidelines published on the IPAQ website [38], the mean PA per week was calculated and is expressed in metabolic equivalent of task (MET)-minutes per week.

### Physical fitness

As a measure of PF, aerobic endurance capacity was assessed by conducting a standard exercise test on a bicycle ergometer (Ergobike medical, Daum, Fürth, Germany). The test was implemented according to the WHO-loading protocol [39], starting at 25 Watt with an increment of 25 Watt every 2 minutes until individual exhaustion. During the test highest achieved oxygen consumption (VO_2 peak_) was assessed by using a cardiopulmonary exercise testing system (MetaMax 3 B, Cortex, Leipzig/Germany). Power at individual anaerobic threshold (P_IAT_) was determined by additional implementation of a lactate test (Biosen C-Line, EKF, Barleben, Germany). Therefore, capillary blood samples were taken from the participant’s earlobe immediately before the test, at the end of every workload level, at the end of the test, and after 1, 3, and 5 minutes of recovery. Determination of P_IAT_ (lactate treshold + 1,0 mmol/l for cycling [40]) was carried out via Ergonizer software (Ergonizer, Version 4, Freiburg, Germany). Prior to the study, break-off criteria were defined and if one of these was reached the test was stopped. The break-off criteria were defined by the study doctor according to national standards for ergometry and recorded in the SOP. Break-off criteria were e.g. ST-segment depression >3mm; ST-segment elevation>1mm, acute hypertension (RR syst. >230mmHG, RR diast >115mmHg), appearance of angina pectoris symptoms or severe dyspnea. For this purpose, we measured the heart rate during the test period by using a heart rate monitor (T31 coded, Polar Electro GmbH Deutschland, Büttelborn, Germany). Throughout the entire procedure hemodynamic monitoring was conducted by running a 12-channel elektrocardiogram (CardioDirect 12, DelMar Reynolds GmbH, Feucht, Germany) and measuring the blood pressure every 2–3 minutes on the right upper arm by using a blood pressure monitor (boso Carat professional, Bosch + Sohn, Jungingen, Germany).

### Statistical methods

For the entire statistical analysis the statistic software SAS JMP 11.0.0 (SAS Institute Inc. 2013, Cary, NC, USA) was used. Data that fell within the normal distribution are shown as mean ± standard deviation (SD). Data that were not normally distributed are shown as median and interquartile range (IQR). For further statistical analysis, variables that were not normaly distributed were log-transformed. For Irisin Box-Cox-Transformation was used. Students t-test was generally used to investigate sex differences for all normally distributed parameters. If data were still not normally distributed after log-transformation, the Wilcoxon test was used. In case variance homogeneity was not given, the Welch correction factor for students t-test or median test instead of Wilcoxon were used. Since body composition and different PA parameters differ enormously between sexes, we also stratified our study group according to sex for further statistical analysis to eliminate the strongest confounder from the very beginning. We used a multivariate linear regression model (MLRA) to investigate associations of the individual PA parameters with Irisin concentrations. Irisin appears to be related to age, FM, LBM and parameters of glucose metabolism in the literature. Therefore, we additionally adjusted the models for these confounders to find the independent influence of PA on the resting Irisin concentration. In order to fulfill the criteria for the MLRA we performed box-cox-transformation of the models and ran outlier diagnostics. The outliers were identified by visual inspection of the predicted versus the studentized residuals plots. Studentized residuals outside the range of -3 and 3 were excluded from future analysis. These plots were also used to ensure homoscedasticity. In total we calculated 12 models for the dependent variable Irisin, 6 for each sex. For each model we focused on one of the 6 PA parameters AEE (kcal/d), TEE (kcal/d), PAL, Total MET (MET- min/week), VO_2 peak_ (l/min), and P_IAT_ (watt) that was adjusted in each case to age (years), FM (%), LBM (g), Glucose (mg/dl), HbA1c (mmol/mol) and Insulin (μlU/ml). In order to evaluate the results, we focused on R^2^ and the significance of the individual models. The latter was calculated by using the F-Test. P-values of the models below 0.05 were considered as statistically significant. The participant numbers for the physical parameters differ due to missing data and because outliers were excluded for further MLRA statistical analysis.

## Results

### Characteristics and sex differences

Table 2 shows the characteristics of 172 men and 128 women participating in the KarMeN study. All participants were non-smokers. Male participants were between 20 and 80 years of age and female participants were 19 to 80 years of age. Statistically significant sex differences were observed for age, body weight, BMI, FM (kg, %), LBM (kg, %), AEE, TEE, VO_2 peak_, P_IAT_ and HbA1c. Men were younger than women and had significantly higher levels of LBM (kg, %), AEE, TEE, VO_2 peak_, and P_IAT_.

**Table 2:** Characteristics of study participants separated by sex.

	Men			Women				
	n	mean/median	SD/IQR	n	mean/median	SD/IQR	p-value ^a^	P
Age (years)*^2^	172	44.1	35.5	128	54.0	21.5	0.0002^†^	
Body weight (kg)*^1^	172	79.2	10.2	128	63.5	11.5	<0.0001^†^	
BMI (kg/m^2^)*^2^	172	24.0	4.3	128	22.7	4.0	<0.0001^†^	
FM (kg)*^2^	172	18.0	10.6	128	21.2	8.4	<0.0001^†^	
FM (%)*^2^*^3^	172	23.6	10.1	128	33.4	6.6	<0.0001^†^	
LBM (kg)*^1^	172	58.0	6.6	128	40.3	3.8	<0.0001^†^	
LBM (%)*^2^*^3^	172	72.5	9.2	128	63.0	6.3	<0.0001^†^	
AEE (kcal/d)*^2^	169	969	675	127	702	332	<0.0001^†^	
TEE (kcal/d)*^2^	169	2827	1023	127	2107	451	<0.0001^†^	
PAL*^2^	169	1.81	0.47	127	1.80	0.31	0.558	0.120
Total MET(MET-min/week)*^2^	172	4205	4012	128	5169	5213	0.168	0.720
VO_2 peak_ (l/min)*^1^*^4^	166	3.37	0.85	114	1.89	0.54	<0.0001^†^	
P_IAT_ (watt)*^1^	164	169.1	41.0	119	107.5	22.1	<0.0001^†^	
Glucose (mg/dl)*^2^	172	86.0	10.0	128	85.0	8.0	0.120	0.744
HbA1c (mmol/mol)*^2^	172	34.4	5.5	128	35.5	5.5	0.019^†^	
Insulin (μlU/ml)*^2^	172	9.3	4.8	128	9.0	5.3	0.210	0.188
Irisin (ng/ml)*^2^	172	9.9	1.8	128	10.2	1.7	0.085	0.592

AEE, activity energy expenditure; BMI, body mass index; FM, fat mass; LBM, lean body mass; MET, metabolic equivalent; PAL, physical activity level; P_IAT_, power at individual anaerobic threshold; TEE, total energy expenditure; VO_2 peak_, peak oxygen consumption; P, Post-Hoc-Analysis for non significant sex differences

*^1^ mean ± SD

*^2^ median ± IQR

*^3^mean ± SD for women

*4 median ± IQR for women

^a^ Difference between men and women

^†^ significant at p < 0.05

### Association between Irisin and physical activity or fitness parameters

Table 3 shows the results of the MLRA for Irisin stratified for men and women. Regression analysis revealed that non of the models were associated with resting Irisin concentrations, neither in men, nor in women. For Irisin the R^2^ was between 0.013 (for P_IAT_ in men) and 0.072 (for AEE in women).

**Table 3:** Multivariate linear regression analysis for Irisin separated by sex.

	Men					Women				
	n	R^2^	p-value	Std. Beta	P	n	R^2^	p-value	Std. Beta	P
Model AEE	166	0.049	0.327		0.520	125	0.072	0.253		0.578
AEE (kcal/d)			0.030	-0.223				0.088	0.166	
Age (years)			0.665	-0.053				0.277	0.131	
FM (%)			0.715	-0.035				0.429	-0.085	
LBM (g)			0.175	0.119				0.409	0.081	
Glucose (mg/dl)			0.254	-0.113				0.210	-0.137	
HbA1c (mmol/mol)			0.487	0.069				0.382	0.093	
Insulin (μlU/ml)			0.304	-0.089				0.066	0.185	
Model TEE	166	0.042	0.449		0.441	126	0.068	0.290		0.549
TEE (kcal/d)			0.061	-0.211				0.030	0.237	
Age (years)			0.698	-0.049				0.340	0.114	
FM (%)			0.770	-0.028				0.549	-0.064	
LBM (g)			0.137	0.142				0.942	0.008	
Glucose (mg/dl)			0.304	-0.102				0.360	-0.099	
HbA1c (mmol/mol)			0.556	0.058				0.453	0.080	
Insulin (μlU/ml)			0.324	-0.085				0.251	0.114	
Model PAL	166	0.047	0.353		0.502	126	0.035	0.743		0.278
PAL			0.035	-0.201				0.426	0.075	
Age (years)			0.749	-0.038				0.554	0.071	
FM (%)			0.704	-0.037				0.785	-0.029	
LBM (g)			0.310	0.085				0.217	0.119	
Glucose (mg/dl)			0.211	-0.125				0.404	-0.092	
HbA1c (mmol/mol)			0.455	0.074				0.445	0.083	
Insulin (μlU/ml)			0.320	-0.086				0.241	0.118	
Model Total MET	169	0.018	0.891		0.191	128	0.030	0.810		0.240
Total MET (MET-min/week)			0.890	0.011				0.380	-0.093	
Age (years)			0.741	0.038				0.773	0.036	
FM (%)			0.721	-0.035				0.904	-0.013	
LBM (g)			0.579	0.046				0.195	0.124	
Glucose (mg/dl)			0.292	-0.106				0.480	-0.078	
HbA1c (mmol/mol)			0.543	0.060				0.305	0.114	
Insulin (μlU/ml)			0.584	-0.047				0.503	0.067	
Model VO_2 peak_	164	0.015	0.935		0.159	114	0.032	0.834		0.226
VO_2 peak_ (l/min)			0.663	-0.057				0.346	0.145	
Age (years)			0.992	-0.001				0.368	0.137	
FM (%)			0.694	-0.041				0.860	0.020	
LBM (g)			0.626	0.048				0.415	0.095	
Glucose (mg/dl)			0.538	-0.061				0.595	-0.062	
HbA1c (mmol/mol)			0.734	0.034				0.549	0.068	
Insulin (μlU/ml)			0.355	-0.080				0.847	0.020	
Model P_IAT_	161	0.013	0.958		0.139	119	0.021	0.931		0.162
P_IAT_ (watt)			0.553	-0.067				0.867	0.021	
Age (years)			0.657	0.052				0.988	-0.002	
FM (%)			0.668	-0.045				0.989	0.002	
LBM (g)			0.420	0.083				0.313	0.109	
Glucose (mg/dl)			0.382	-0.089				0.492	-0.078	
HbA1c (mmol/mol)			0.710	0.038				0.490	0.078	
Insulin (μlU/ml)			0.701	-0.034				0.509	0.068	

AEE: activity energy expenditure; P_IAT_: power at individual anaerobic threshold; MET: metabolic equivalent; PAL: physical activity level; TEE: total energy expenditure; VO_2 peak_: maximal oxygen consumption; FM: fat mass; LBM: lean body mass; P: Post-Hoc-Analysis

## Discussion

The current study served to investigate the association between resting Irisin concentrations and regular PA and PF status in serum of healthy humans. Our data show that none of the determined PA parameters comprising AEE, TEE, PAL, Total MET, VO_2 peak_, and P_IAT_ are associated with resting Irisin serum concentration, suggesting that PA level and PF status are not related to resting Irisin concentrations in healthy humans.

Acute exercise increases Irisin blood concentration [20–29]. Data from the association between habitual PA [20, 30] or PF [25, 26, 31, 32] with resting Irisin concentrations are still inconsistent and additionally less well investigated.

In our study we applied three different activity assessments and categorized the outcome parameters into three different groups: short-term PA, habitual PA, and PF in order to obtain a comprehensive evaluation of the PA status and PF and finally to examine the relationship between these and the resting Irisin concentration in healthy humans. Furthermore, we calculated data separated by sex to eliminate the strongest confounder from the very beginning.

In line with our results other studies did not find a significant relationship between the resting Irisin concentration and PF in healthy subjects [26, 32], whereas Daskalopoulou and colleagues did not separate the study group according to sex in their statistical analysis. In contrast Kerstholt and colleagues found an inverse association between the Irisin concentration and the two PA parameters VO_2 peak_ and Pmax in 322 men, whereas a significant positive association between VO_2 peak_ could be found in a subgroup of 418 women, after excluding those whose time between VO_2 peak_ determination and blood collection was too large [31]. We also separated our study group for statistical analysis and could not find any association between the resting Irisin concentration and VO_2 peak_ in both sexes. Consistent with the results of Kerstholt and colleagues, others found a lower baseline Irisin concentration in physically active compared to sedentary male subjects [25]. Compared to Huh and colleagues, we used multivariate linear regression analysis instead of separating our study population in active and sedentary groups, to eliminate the bias of marginal groups.

Similar to our results Anastasilakis and colleagues also did not find a statistically significant difference between habitual PA levels and resting Irisin in healthy young subjects by using a modified version of the IPAQ and separating the study population into low, moderate, or high physical active groups [20]. In contrast, a weak association could be shown between habitual PA and Irisin concentration in healthy controls using a standardized questionnaire with the frequency intensity time index as outcome parameter [41]. However, we decided to assess the habitual PA level by using the long-form of the IPAQ, since valid and reliable PA data from 18 to 65 year old adults can be collected using this instrument [42]. Furthermore, we separated our study group according to sex to reduce sex specific influences. In order to determine the level of short-term PA, we used an accelerometer and also could not find any association between the short-term PA level and the resting Irisin concentration. In contrast, moderate and high intensity ambulatory activity levels were significantly positively associated with Irisin concentrations in a study population consisting of sedentary lean (healthy), obese, prediabetes, and Type 2 diabetes men [30]. Unfortunately, the authors did not exclusively focus on the sedentary (healthy) lean subjects in their multiple linear regression analysis. That makes it difficult to compare the results to ours, since our study focused on data of healthy participants. Subjects with diabetes type-2 for example seem to have lower baseline Irisin blood concentrations [30, 43, 44]. However, the different time frame of wearing the accelerometer should also be considered, as the KarMeN subjects had to wear the accelerometer for a period of seven continuous days compared to three days in the other study. To our knowledge there is no suggestion for an optimal time frame of wearing an accelerometer. Nevertheless, in order to get valid data, a seven day period including a weekday and a weekend has been suggested [45].

In our study we focused on the association between the regular PA or PF status and resting Irisin concentration in healthy humans without conducting any intervention. Results from exercise interventions [22, 24, 29, 30, 32–36, 46, 47] are therefore not comparable to ours.

As we expected, the sex differences for all body composition parameters and 4 of 6 PA parameters were statistically significant, confirming the necessity of separated calculations for further statistical analysis and moreover for the accuracy of data. Focusing on the differences in resting Irisin blood concentration, no sex differences were found in our study. Consistent with our results, other studies also did not find a sex difference in healthy subjects [31] or healthy controls [48], while one study showed lower Irisin concentrations in males after adjusting for LBM [20].

## Conclusion

We performed our study with a large population number of 300 participants and applied three different PA assessment instruments (IPAQ, Accelerometer, and cardiopulmonary exercise testing with additional implementation of lactate test) under strictly controlled conditions using standardized protocols. Thus we were able to investigate the influence of short-term PA, habitual PA, and PF status on resting Irisin serum concentration. Our data clearly show that none of the PA parameters representing short-term PA, habitual PA, as well as PF are associated with resting Irisin serum concentrations in healthy humans. Therefore we assume that if PA related effects are partially induced by myokines, permanently increased Irisin serum concentrations may not be necessary to induce health-related exercise effects. Of course, further research will have to be done to get a better understanding of the association between the resting Irisin concentration and the PA or PF status.
